# Supplementary material for: The molecular appearance of native TRPM7 channel complexes identified by high-resolution proteomics
Source: eLife. 2021 Nov 12;10:e68544. doi: 10.7554/eLife.68544 (PMC8616561; doi:10.7554/eLife.68544)
Supplement: Supplementary file 3. — Excel file contains one worksheet: The phosphorylated residues of TRPM7, CNNM3, and CNNM4 identified by mass spectrometry (MS) in the present study are outlined in conjunction with previously published data (Nguyen et al., 2019; Zhou et al., 2013; Cai et al., 2017; Huttlin et al., 2010). [file elife-68544-supp3.docx]

**TRPM7 phosphosites identified in samples from HEK293 cells**

**S101:** n = 1

| Score | Mr(calc) | Delta | Sequence | Site Analysis |
| --- | --- | --- | --- | --- |
| 45.5 | 2630.1187 | -0.0015 | HTEQSPTDAYGVINFQGGSHSYR | Phospho S19 75.16% |

**S554:** n = 2

| Score | Mr(calc) | Delta | Sequence | Site Analysis |
| --- | --- | --- | --- | --- |
| 27.6 | 1169.5078 | -0.0003 | NTSSSTPQLR | Phospho S5 74.06% |

**S561:** n = 1

| Score | Mr(calc) | Delta | Sequence | Site Analysis |
| --- | --- | --- | --- | --- |
| 26.6 | 1026.3920 | -0.0006 | SHETFGNR | Phospho S1 93.33% |

**S1208:** n = 1

| Score | Mr(calc) | Delta | Sequence |
| --- | --- | --- | --- |
| 37.5 | 1154.5406 | -0.0002 | VEQMSIQIK |

**S1230:** n = 1

| Score | Mr(calc) | Delta | Sequence | Site Analysis |
| --- | --- | --- | --- | --- |
| 62.8 | 2661.3262 | 0.0017 | SLQSLDSQIGHLQDLSALTVDTLK | Phospho S7 74.52% |

**S1255:** n = 5

| Score | Mr(calc) | Delta | Sequence | Site Analysis |
| --- | --- | --- | --- | --- |
| 69.2 | 1313.6228 | -0.0013 | TLTAQKASEASK | Phospho S8 98.68% |

**S1258:** n = 2

| Score | Mr(calc) | Delta | Sequence | Site Analysis |
| --- | --- | --- | --- | --- |
| 45.9 | 1520.6984 | 0.0011 | ASEASKVHNEITR | Phospho S5 84.38% |

**S1255 + S1258:** n = 1

| Score | Mr(calc) | Delta | Sequence | Site Analysis |
| --- | --- | --- | --- | --- |
| 40.3 | 1600.6647 | 0.0007 | ASEASKVHNEITR | Phospho S2, S5 99.97% |

**S1269:** n = 2

| Score | Mr(calc) | Delta | Sequence | Site Analysis |
| --- | --- | --- | --- | --- |
| 28.0 | 755.3466 | -0.0004 | ELSISK | Phospho S3 68.98% |

**S1271:** n = 2

| Score | Mr(calc) | Delta | Sequence | Site Analysis |
| --- | --- | --- | --- | --- |
| 24.9 | 755.3466 | -0.0006 | ELSISK | Phospho S5 81.50% |

**T1296:** n = 1

| Score | Mr(calc) | Delta | Sequence | Site Analysis |
| --- | --- | --- | --- | --- |
| 30.7 | 1835.8778 | -0.0029 | KPSAVNTLSSSLPQGDR | Phospho T7 61.77% |

**S1300:** n = 6

| Score | Mr(calc) | Delta | Sequence | Site Analysis |
| --- | --- | --- | --- | --- |
| 111.2 | 1835.8778 | 0.0010 | KPSAVNTLSSSLPQGDR | Phospho S11 92.73% |

**S1360:** n = 3

| Score | Mr(calc) | Delta | Sequence | Site Analysis |
| --- | --- | --- | --- | --- |
| 40.4 | 2711.2302 | 0.0058 | EFNIPEAGSSCGALFPSAVSPPELR | Phospho S20 92.52% |

**S1385:** n = 3

| Score | Mr(calc) | Delta | Sequence | Site Analysis |
| --- | --- | --- | --- | --- |
| 79.1 | 1789.7706 | -0.0044 | LGSSPNSSPHMSSPPTK | Phospho S3 97.08% |

**S1386:** n = 2

| Score | Mr(calc) | Delta | Sequence | Site Analysis |
| --- | --- | --- | --- | --- |
| 52.0 | 1789.7706 | 0.0001 | LGSSPNSSPHMSSPPTK | Phospho S4 63.00% |

**S1386 + S1389:** n = 3

| Score | Mr(calc) | Delta | Sequence | Site Analysis |
| --- | --- | --- | --- | --- |
| 32.6 | 1869.7369 | -0.0037 | LGSSPNSSPHMSSPPTK | Phospho S4, S7 97.03% |

**S1389:** n = 1

| Score | Mr(calc) | Delta | Sequence | Site Analysis |
| --- | --- | --- | --- | --- |
| 48.4 | 1789.7706 | -0.0008 | LGSSPNSSPHMSSPPTK | Phospho S7 88.90% |

**S1389 + S1394:** n = 1

| Score | Mr(calc) | Delta | Sequence | Site Analysis |
| --- | --- | --- | --- | --- |
| 21.8 | 1869.7369 | -0.0001 | LGSSPNSSPHMSSPPTK | Phospho S8, S12 30.77% |

**S1394:** n = 1 (S1394 or S1395 not unambiguous)

| Score | Mr(calc) | Delta | Sequence | Site Analysis |
| --- | --- | --- | --- | --- |
| 40.4 | 1789.7706 | 0.0016 | LGSSPNSSPHMSSPPTK | Phospho S12 46.49% |

**S1395:** n = 2 (detected in HEK293 WT only)

| Score | Mr(calc) | Delta | Sequence | Site Analysis |
| --- | --- | --- | --- | --- |
| 41.6 | 1774.8502 | -0.0008 | LGSSSTSIPHLSSPPTK | Phospho S13 74.92% |

**S1403:** n = 3

| Score | Mr(calc) | Delta | Sequence | Site Analysis |
| --- | --- | --- | --- | --- |
| 46.0 | 1403.5792 | -0.0016 | FSVSTPSQPSCK | Phospho S4 76.17% |

**S1445:** n = 4

| Score | Mr(calc) | Delta | Sequence |
| --- | --- | --- | --- |
| 57.8 | 2614.1272 | 0.0057 | AAEGDNIEFGAFVGHRDSMDLQR |

**T1466:** n = 3

| Score | Mr(calc) | Delta | Sequence | Site Analysis |
| --- | --- | --- | --- | --- |
| 64.3 | 1552.7021 | -0.0027 | ELLSNDTPENTLK | Phospho T7 99.96% |

**S1480:** n = 1

| Score | Mr(calc) | Delta | Sequence | Site Analysis |
| --- | --- | --- | --- | --- |
| 49.4 | 1417.5156 | 0.0011 | HVGAAGYSECCK | Phospho S8 100.00% |

**T1485:** n = 1

| Score | Mr(calc) | Delta | Sequence | Site Analysis |
| --- | --- | --- | --- | --- |
| 30.5 | 3048.2525 | -0.0012 | HVGAAGYSECCKTSTSLHSVQAESCSR | Phospho T13 31.86% |

**T1485 + T1498:** n = 2

| Score | Mr(calc) | Delta | Sequence | Site Analysis |
| --- | --- | --- | --- | --- |
| 34.1 | 1808.6801 | -0.0027 | TSTSLHSVQAESCSR | Phospho T1, S14 96.71% |

**S1488:** n = 4

| Score | Mr(calc) | Delta | Sequence | Site Analysis |
| --- | --- | --- | --- | --- |
| 70.5 | 1728.7138 | -0.0002 | TSTSLHSVQAESCSR | Phospho S4 66.92% |

**S1488 + S1491:** n = 2

| Score | Mr(calc) | Delta | Sequence | Site Analysis |
| --- | --- | --- | --- | --- |
| 42.3 | 1808.6801 | 0.0009 | TSTSLHSVQAESCSR | Phospho S4, S7 99.19% |

**S1491:** n = 1

| Score | Mr(calc) | Delta | Sequence | Site Analysis |
| --- | --- | --- | --- | --- |
| 68.8 | 1728.7138 | -0.0006 | TSTSLHSVQAESCSR | Phospho S7 82.50% |

**S1491 + S1496:** n = 1

| Score | Mr(calc) | Delta | Sequence | Site Analysis |
| --- | --- | --- | --- | --- |
| 19.1 | 1808.6801 | -0.0008 | TSTSLHSVQAESCSR | Phospho S7, S12 81.63% |

**S1496:** n = 2

| Score | Mr(calc) | Delta | Sequence | Site Analysis |
| --- | --- | --- | --- | --- |
| 43.9 | 1728.7138 | -0.0024 | TSTSLHSVQAESCSR | Phospho S12 87.23% |

**S1498:** n = 2

| Score | Mr(calc) | Delta | Sequence | Site Analysis |
| --- | --- | --- | --- | --- |
| 49.7 | 1728.7138 | -0.0020 | TSTSLHSVQAESCSR | Phospho S14 87.03% |

**S1502:** n = 3

| Score | Mr(calc) | Delta | Sequence | Site Analysis |
| --- | --- | --- | --- | --- |
| 74.1 | 1499.6141 | -0.0008 | RASTEDSPEVDSK | Phospho S3 92.83% |

**S1502 + S1506:** n = 1

| Score | Mr(calc) | Delta | Sequence | Site Analysis |
| --- | --- | --- | --- | --- |
| 62.6 | 1579.5804 | 0.0008 | RASTEDSPEVDSK | Phospho S3, S7 94.51% |

**S1506:** n = 3

| Score | Mr(calc) | Delta | Sequence | Site Analysis |
| --- | --- | --- | --- | --- |
| 60.9 | 1499.6141 | -0.0020 | RASTEDSPEVDSK | Phospho S7 99.52% |

**S1565:** n = 1

| Score | Mr(calc) | Delta | Sequence | Site Analysis |
| --- | --- | --- | --- | --- |
| 38.3 | 1515.7851 | 0.0019 | LSQSIPFVPVPPR | Phospho S2 77.41% |

**S1567:** n = 6

| Score | Mr(calc) | Delta | Sequence | Site Analysis |
| --- | --- | --- | --- | --- |
| 41.7 | 1517.7643 | -0.0003 | LSQSIPFTPVPPR | Phospho S4 85.76% |

**S1613:** n = 4

| Score | Mr(calc) | Delta | Sequence |
| --- | --- | --- | --- |
| 42.9 | 1660.7531 | -0.0031 | IEFLSKEEMGGGLR |

**S1839:** n = 1

| core | Mr(calc) | Delta | Sequence | Site Analysis |
| --- | --- | --- | --- | --- |
| 52.7 | 2272.0260 | 0.0007 | IIFPQDESSDLNLQSGNSTK | Phospho S8 77.33% |

**S1846:** n = 1

| Score | Mr(calc) | Delta | Sequence | Site Analysis |
| --- | --- | --- | --- | --- |
| 42.9 | 2272.0260 | -0.0005 | IIFPQDESSDLNLQSGNSTK | Phospho S15 91.04% |

**S1849:** n = 2

| Score | Mr(calc) | Delta | Sequence | Site Analysis |
| --- | --- | --- | --- | --- |
| 41.3 | 2272.0260 | 0.0013 | IIFPQDESSDLNLQSGNSTK | Phospho S18 80.27% |

**S1853:** n = 1

| Score | Mr(calc) | Delta | Sequence | Site Analysis |
| --- | --- | --- | --- | --- |
| 47.3 | 3245.4725 | -0.0097 | IIFPQDESSDLNLQSGNSTKESEATNSVR | Phospho S22 77.96% |

**TRPM7 phosphosites identified in mouse brain samples**

**S1255:** n = 9

| Score | Mr(calc) | Delta | Sequence | Site Analysis |
| --- | --- | --- | --- | --- |
| 59.8 | 1313.6228 | -0.0025 | TLTAQKASEASK | Phospho S8 87.74% |

**S1300:** n = 2 (S1300 or S1299 not unambiguous)

| Score | Mr(calc) | Delta | Sequence | Site Analysis |
| --- | --- | --- | --- | --- |
| 27.0 | 1835.8778 | 0.0016 | KPSAVNTLSSSLPQGDR | Phospho S11 48.73% |

**S1360:** n = 4

| Score | Mr(calc) | Delta | Sequence | Site Analysis |
| --- | --- | --- | --- | --- |
| 48.1 | 2711.2302 | 0.0001 | EFNIPEAGSSCGALFPSAVSPPELR | Phospho S20 80.06% |

**S1386:** n = 3

| Score | Mr(calc) | Delta | Sequence | Site Analysis |
| --- | --- | --- | --- | --- |
| 49.2 | 1789.7706 | -0.0002 | LGSSPNSSPHMSSPPTK | Phospho S4 69.58% |

**S1403:** n = 3

| Score | Mr(calc) | Delta | Sequence | Site Analysis |
| --- | --- | --- | --- | --- |
| 38.3 | 1403.5792 | -0.0010 | FSVSTPSQPSCK | Phospho S4 84.05% |

**T1466:** n = 4

| Score | Mr(calc) | Delta | Sequence | Site Analysis |
| --- | --- | --- | --- | --- |
| 48.0 | 1821.8873 | 0.0002 | IRELLSNDTPENTLK | Phospho T9 98.30% |

**S1488:** n = 1

| Score | Mr(calc) | Delta | Sequence | Site Analysis |
| --- | --- | --- | --- | --- |
| 51.4 | 1728.7138 | 0.0006 | TSTSLHSVQAESCSR | Phospho S4 36.29% |

**S1502:** n = 5

| Score | Mr(calc) | Delta | Sequence | Site Analysis |
| --- | --- | --- | --- | --- |
| 69.8 | 1499.6141 | -0.0016 | RASTEDSPEVDSK | Phospho S3 96.02% |

**S1567:** n = 3

| Score | Mr(calc) | Delta | Sequence | Site Analysis |
| --- | --- | --- | --- | --- |
| 28.1 | 1515.7851 | 0.0032 | LSQSIPFVPVPPR | Phospho S4 70.77% |

**CNNM3 phosphosites identified in samples from HEK293 cells**

**S700:** n = 1

| Score | Mr(calc) | Delta | Sequence | Site Analysis |
| --- | --- | --- | --- | --- |
| 62.1 | 2554.1926 | -0.0024 | [TTTAAGSSHSRPGVPVEGSPGRNPGV](http://mascot.physiol2.intra.uni-freiburg.de/mascot/cgi/peptide_view.pl?file=v%3A%2FMascot%2FResults%2F20200312%2FF133647.dat&hit=1&px=1&query=19373&section=5&ave_thresh=23&report=&_sigthreshold=&_msresflags=3138&_msresflags2=266&percolate=&percolate_rt=) | Phospho S19 99.92% |

**CNNM4 phosphosites identified in samples from HEK293 cells**

**S664:** n = 1

| Score | Mr(calc) | Delta | Sequence | Site Analysis |
| --- | --- | --- | --- | --- |
| 20.4 | 1074.4383 | -0.0008 | [SASLSYPDR](http://mascot.physiol2.intra.uni-freiburg.de/mascot/cgi/peptide_view.pl?file=v%3A%2FMascot%2FResults%2F20191006%2FF127139.dat&hit=1&px=1&query=4394&section=5&ave_thresh=26&report=&_sigthreshold=&_msresflags=3138&_msresflags2=266&percolate=&percolate_rt=) | Phospho S3 80.54% |

n indicates the number of peptide spectra identifying phosphorylation at the same site.

The table gives Mascot-Score of the identified peptide (Score), calculated molecular weight in Dalton (Mr(calc)), deviation of the measured form the calculated molecular weight in Dalton (Delta), amino acid sequence of the assigned peptide in single letter code (sequence) and likelihood that phosphorylation was assigned to the correct site within the peptide sequence (site analysis).
