# Supplementary material for: The molecular appearance of native TRPM7 channel complexes identified by high-resolution proteomics
Source: eLife. 2021 Nov 12;10:e68544. doi: 10.7554/eLife.68544 (PMC8616561; doi:10.7554/eLife.68544)
Supplement: Transparent reporting form [file elife-68544-transrepform1.docx]

***­­­­­­­­***

***eLife’s* transparent reporting form**

We encourage authors to provide detailed information *within their submission* to facilitate the interpretation and replication of experiments. Authors can upload supporting documentation to indicate the use of appropriate reporting guidelines for health-related research (see [EQUATOR Network](http://www.equator-network.org/%20)), life science research (see the [BioSharing Information Resource](https://biosharing.org/)), or the [ARRIVE guidelines](http://www.plosbiology.org/article/info:doi/10.1371/journal.pbio.1000412) for reporting work involving animal research. Where applicable, authors should refer to any relevant reporting standards documents in this form.

If you have any questions, please consult our Journal Policies and/or contact us: [editorial@elifesciences.org](mailto:editorial@elifesciences.org).

**Sample-size estimation**

- You should state whether an appropriate sample size was computed when the study was being designed
- You should state the statistical method of sample size computation and any required assumptions
- If no explicit power analysis was used, you should describe how you decided what sample (replicate) size (number) to use

Please outline where this information can be found within the submission (e.g., sections or figure legends), or explain why this information doesn’t apply to your submission:

Membrane fractions from mouse or rat brains were prepared from 25 male and female animals each to avoid biases introduced by individual animals. This information is given in the methods section.

A total of 17 commercially obtained or homemade antibodies targeting TRPM7 was tested for their efficiency to precipitate TRPM7. Only the most efficient of these antibodies were used for preparative affinity purifications. For preparative scale affinity purifications each individual antibody was used once with each membrane preparation and under each solubilisation condition described, and purified proteins analysed for their consistent co-purification with at least two of three or four different antibodies under the same condition as described in the results section.

In electrophysiological experiments with *Xenopus laevis* oocytes (Figure 3, Figure 3-figure supplement 1-3, Figure 4, Figure 4-figure supplement 2, Figure 6) and HEK293 cells (Figure 3-figure supplement 4, Figure 4-figure supplement 1), no sample-size estimation and power analysis were performed because of an unknown outcome of the experiments. The number of examined oocytes and HEK293 cells were reported in the corresponding figure legends.

**Replicates**

- You should report how often each experiment was performed
- You should include a definition of biological versus technical replication
- The data obtained should be provided and sufficient information should be provided to indicate the number of independent biological and/or technical replicates
- If you encountered any outliers, you should describe how these were handled
- Criteria for exclusion/inclusion of data should be clearly stated
- High-throughput sequence data should be uploaded before submission, with a private link for reviewers provided (these are available from both GEO and ArrayExpress)

Please outline where this information can be found within the submission (e.g., sections or figure legends), or explain why this information doesn’t apply to your submission:

Number of technical replicates is mentioned in figure legends where applicable.

Quantification of proteins in APs and csBNMS were based on at least 2 and 3 protein-specific peptides, respectively (see methods).

The number of spectral matches underlying each of the verified phosphorylation sites is given together with the reference spectra in supplementary file 2 to figure 6.

In the experiments with *Xenopus laevis* oocytes and HEK293 cells, the definition of biological versus technical replicates (i.e. number of oocytes, blots, fluorescence images) is indicated in the corresponding figure legends.

**Statistical reporting**

- Statistical analysis methods should be described and justified
- Raw data should be presented in figures whenever informative to do so (typically when N per group is less than 10)
- For each experiment, you should identify the statistical tests used, exact values of N, definitions of center, methods of multiple test correction, and dispersion and precision measures (e.g., mean, median, SD, SEM, confidence intervals; and, for the major substantive results, a measure of effect size (e.g., Pearson's r, Cohen's d)
- Report exact p-values wherever possible alongside the summary statistics and 95% confidence intervals. These should be reported for all key questions and not only when the p-value is less than 0.05.

Please outline where this information can be found within the submission (e.g., sections or figure legends), or explain why this information doesn’t apply to your submission:

For the experiments with *Xenopus laevis* oocytes (Figure 3, Figure 3-figure supplement 1-3, Figure 4, Figure 4-figure supplement 2, Figure 6) and HEK293 cells (Figure 3-figure supplement 4, Figure 4-figure supplement 1,2) the corresponding figure legends indicate the statistical test used, exact values of n, value of raw data (mean+/-SEM) and p-values.

(For large datasets, or papers with a very large number of statistical tests, you may upload a single table file with tests, Ns, etc., with reference to sections in the manuscript.)

**Group allocation**

- Indicate how samples were allocated into experimental groups (in the case of clinical studies, please specify allocation to treatment method); if randomization was used, please also state if restricted randomization was applied
- Indicate if masking was used during group allocation, data collection and/or data analysis

Please outline where this information can be found within the submission (e.g., sections or figure legends), or explain why this information doesn’t apply to your submission:

Affinity purifications from different antibodies with the same membrane fraction and under the same solubilisation condition were allocated to one experimental group as described in the results section.

**Additional data files (“source data”)**

- We encourage you to upload relevant additional data files, such as numerical data that are represented as a graph in a figure, or as a summary table
- Where provided, these should be in the most useful format, and they can be uploaded as “Source data” files linked to a main figure or table
- Include model definition files including the full list of parameters used
- Include code used for data analysis (e.g., R, MatLab)
- Avoid stating that data files are “available upon request”

Please indicate the figures or tables for which source data files have been provided:

MS raw data related to figure 1 are available via ProteomeXchange with identifier PXD025279.

Quantitative MS data related to figure 1 and table 1 (peptides LC-MS signal intensities (peak volumes, PVs), protein abundance ratios, target- and background normalized abundance ratios (= ratio distance values) and abundancenorm values (as measures for absolute abundance), all determined as described in methods) are provided in Excel format (supplementary file 1 to figure 1 and table 1).

Exemplary phosphopeptide spectra for each identified phosphorylation site are provided in supplementary file 2 to figure 6.

Phosphorylation sites in TRPM7, CNNM3 and CNNM4 identified in APs from transfected HEK293 cells and rodent brain are provided in supplementary file 3 to figure 6.
